# In Utero Alcohol and Tobacco Exposure, Maternal Depression, And Maternal Obesity Are Associated with Impaired Oligodendrocyte Differentiation in The Developing Brain

**DOI:** 10.26502/ogr0172

**Published:** 2025-01-15

**Authors:** Uday Bharai, Jamal Hamze, Benjamin Zhang, Monica Hampe, Emily Sparks, Nana Merabova, Gabriel Tatevosian, Armine Darbinyan, Mary F Morrison, Laura Goetzl, Nune Darbinian, Michael Selzer

**Affiliations:** 1 Center for Neural Development and Repair, Department of Neural Sciences, Lewis Katz School of Medicine at Temple University, Philadelphia, PA 19140, USA.; 2 Department of Obstetrics and Gynecology, Gundersen Health System, La Crosse, WI 54601, USA.; 3 Department of Pathology, Yale University School of Medicine, New Haven, CT 06520, USA.; 4 Department of Psychiatry, Lewis Katz School of Medicine at Temple University, Philadelphia, PA 19140, USA.; 5 Department of Obstetrics, Gynecology and Reproductive Sciences, McGovern Medical School at The University of Texas Health Science Center at Houston (UTHealth), Houston, TX 77030, USA.

**Keywords:** Alcohol, Tobacco, FASD, Oligodendrocytes, MBP, Obesity, Depression, Exosomes

## Abstract

**Introduction:**

Fetal alcohol spectrum disorder (FASD) is the leading preventable cause of pediatric cognitive disability and is associated with dysmyelination. We examined possible clinical co-determinants that might interact with EtOH in impairing oligodendrocyte (OL) development. Women who drink, including pregnant women, also disproportionately suffer from depression (mDepression), which we have shown is a risk factor for FASD. Might depression during pregnancy contribute to OL pathology? Maternal obesity (mObesity) also inhibits white matter development in fetal brain. Finally, tobacco exposure inhibits not only OL development, but also the production of structural proteins, such as actin. Our human biobank derived from voluntarily terminated pregnancies allows us to study the effect of EtOH and tobacco exposure, mDepression and mObesity on OL markers.

**Methods:**

Fetal brain tissue (10 – 22 weeks) was collected and EtOH exposure estimated, based on a questionnaire adapted from the NIAAA PASS study. EtOH, tobacco, mObesity, mDepression exposed samples were compared with controls matched for gestational age and fetal gender. RNA expressions of OL markers were assayed by ddPCR. Fetal-brain-derived exosomes (FB-E) were isolated from maternal plasma. Exosomal RNA was studied for MBP, BDNF and actin mRNA expression by qRT-PCR and protein levels were confirmed by ELISA.

**Results:**

Forty-two subjects were used in EtOH, mObesity and mDepression studies, 40 cases were used in EtOH and tobacco studies, and 40 cases were used in OL-E (oligodendrocyte-derived exosomes) studies. Six cases were compared to 6 controls. EtOH exposure, mDepression and mObesity were associated with reduced mRNA expression of myelin basic **protein (MBP)**, a marker for mature OLs: ↓ 1.6-fold with EtOH, ↓ 1.5 mObesity, and ↓ 2.2 mDepression. The combination of EtOH and mObesity was associated with strong reductions in MBP expression (↓ 20.6), as was mDepression plus mObesity (↓ 2.6). No significant effects were observed for the early OL marker **Nkx2.2** (↓ 1.06). **Olig1** was reduced in single (↓ 1.85 EtOH, ↓ 1.8 mObesity) or combined groups: ↓ 5 EtOH and mDepression, ↓ 6.4 EtOH and mObesity, and ↓ 11.6 mDepression and mObesity. We observed reduced **Olig2** (↓1.1 EtOH, ↓ 29 mDepression) in all combined groups. EtOH and mDepression, and obesity were associated with much lower levels of **BDNF** (↓ 1.7 EtOH, ↓ 99). In FB-E studies, 10 cases (EtOH, Tobacco, or EtOH plus Tobacco) were compared to 10 controls: EtOH exposure, Tobacco exposure and EtOH plus Tobacco exposures were associated with reduced **MBP:** ↓ 1.8-fold by EtOH, ↓ 2.6 EtOH plus Tobacco, ↓ 1.9 Tobacco. EtOH and Tobacco had strong inhibitory effect also on **BDNF** (↓2.6), as well as on **Actin** (↓3.9). Cases with high BMI were associated with a stronger effect on MBP downregulation compared to low BMI.

**Conclusions:**

Single Exposures to EtOH or tobacco, mObesity and mDepression all are associated with delayed OL maturation. When these exposures are combined the effects appear to be synergistic. Our unique biobank can be used to determine the mechanism(s) of specific adverse exposures and may suggest novel therapeutic or prophylactic interventions to lessen the severity of FASD.

## 1. Introduction:

Prenatal exposure to alcohol (PAE) can lead to preventable cognitive, behavioral, physical disabilities in children [1]. Fetal alcohol spectrum disorder (FASD) is the leading preventable cause of pediatric cognitive disability and is associated with dysmyelination [2]. The toxic effects of in utero exposure to alcohol on fetal brain development has been discussed extensively, and several biomarkers for early detection of FASD have been proposed [3, 4, 5]. Similarly, cigarette smoke exposure during pregnancy negatively affects brain growth and development, which produces functional disturbance that are apparent in both short- (in infancy) and long-term (into adulthood). This effect of exposure to tobacco is associated with inhibition of brain derived neurotrophic factor (BDNF) expression in the brains of rat pups of dams exposed to two cigarettes twice daily for six weeks [7]. Tobacco exposure inhibits not only OL development, but also the production of structural proteins, such as actin. Because in humans, a blinding condition called tobacco-alcohol amblyopia appears to result from synergistic action between tobacco smoking and chronic alcohol abuse, although it is still not clear whether one substance is primary and the other an aggravating factor [8 9], we postulated that tobacco and alcohol use might act synergistically in the pathogenesis of FASD. Independent of alcohol consumption, prenatal tobacco exposure poses significant risks to pregnancy and childbirth, including low birth weight and preterm delivery - complications also observed in FASD [6]. Cigarette smoking remains another leading cause of preventable disease and death in the United States [10] and kills more than 480,000 Americans each year. In 2021, an estimated 11.5% (28.3 million) of U.S. adults currently smoked cigarettes. Combined prenatal exposure to tobacco and alcohol has been shown to synergistically exacerbate these outcomes [6]. We wanted to see whether a similar synergistic effect extends to the dysmyelination in FASD by examining OL-specific fetal brain-derived exosome markers. On the other hand, maternal obesity (mObesity) has been shown to negatively affect the myelination of fetal neurons [11]. Finally, women who drink, including pregnant women, also disproportionately suffer from depression, which we have shown is a risk factor for FASD [5]. Thus, depression during pregnancy might contribute to OL pathology. Considering this, we wanted to explore whether there is synergy between mObesity and PAE on the severity of FASD. In the present study, we focused primarily on defining the alcohol and tobacco exposure-associated changes in the protein expression of the OL marker, myelin basic protein (MBP) in OL-specific fetal brain-derived exosomes (FB-E). Previously, we showed that FB-E cross the placenta and can be isolated non-invasively from maternal blood. MBP is a vital constituent of both central nervous system (CNS) and peripheral nervous system (PNS) myelin. Previously, it was thought that MBP in cerebrospinal fluid (CSF) may provide information about the status of CNS myelin damage. While MBP normally is not detectable in CSF, following an acute relapse of the demyelinating disease multiple sclerosis (MS), CSF MBP levels rise transiently in the range of ng/ml. Thus, detection of MBP levels in FB-E early in alcohol-exposed pregnancy, might be a promising tool for early diagnosis of FASD. We hypothesized that maternal exposure to tobacco and/or alcohol would alter levels of MBP compared to gestational age-matched controls. Changes in MBP would also suggest that women exposed to tobacco or alcohol might have increased fetal exposure to other drugs.

Our human biobank derived from voluntarily terminated pregnancies allows us to study the effect of EtOH and tobacco exposure, as well as maternal characteristics such as mDepression and mObesity, on OL markers.

## 2. Results

### 2.1. EtOH exposure in combination with mObesity and mDepression is associated with a decrease in expression of OL late markers in fetal brain.

**Figure 1.**
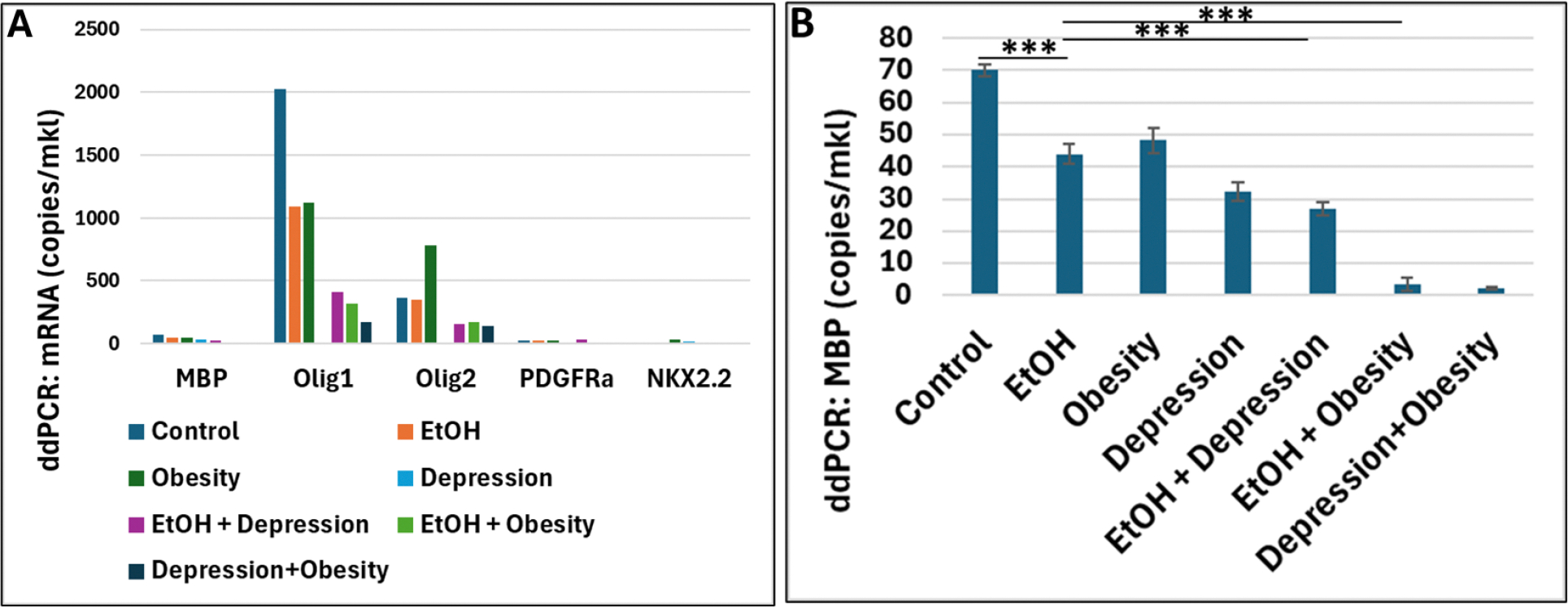
Effects of combinations of risk factors on expression levels of OL lineage markers in fetal brain. **A.** Effects of combinations of EtOH exposure, mObesity, and mDepression, compared with unexposed controls (n=6 fetal brains for each group) on OL early and late markers mRNA expression measured by ddPCR. **B.** Downregulation of the MBP mRNA expression in EtOH-, mObesity-, and depression-exposed fetal brains, alone or in combinations (n=6 for each group). Data are presented as fold change. *** p < 0.001.

To study the effects of combinations of EtOH exposure, mObesity, and mDepression on the mRNA expression levels of early and late OL lineage markers, fetal brains from subjects exposed to EtOH, with or without mObesity and mDepression (Table 1A) were used in ddPCR assays (Figure 1). EtOH exposure, mDepression and mObesity were associated with inhibition of MBP and Olig1, while Olig2 and PDGFRa were affected only by mDepression, and effect on NKX2.2 expression was opposite to the effects on late markers, mostly over-expressed in all conditions (Figure 1A). MBP mRNA expression was significantly more downregulated in all combinations, such as EtOH plus mDepression, EtOH plus mObesity, or mDepression plus mObesity (Figure 1B).

### 2.2. Effects of in utero EtOH and tobacco exposure on MBP and BDNF expression in human FB-Es. Next assays were performed for FB-Es.

**Figure 2.**
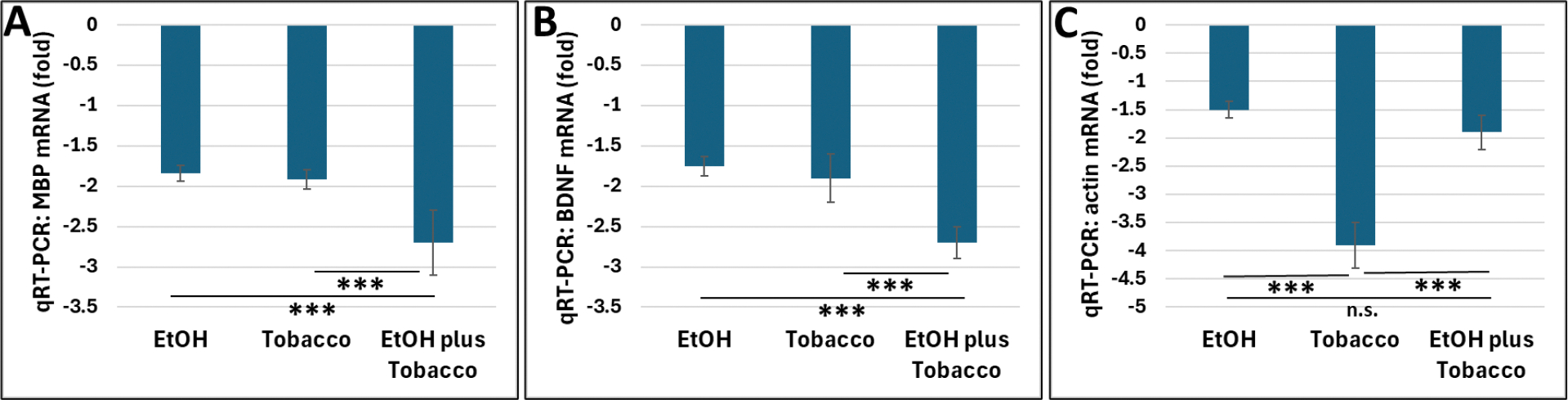
Effects of in utero EtOH and tobacco exposure on MBP and BDNF expression in human FB-Es. **A.** Downregulation of MBP levels in qRT-PCR assays of RNA samples, comparing 10 1^st^ and 2^nd^ trimester EtOH- and tobacco-exposed human FB-Es with 10 unexposed controls individually matched for GA and BMI. **B.** Reduced levels of BDNF in the same FB-Es. **C.** Downregulation of actin in tobacco-exposed FB-Es. Data are presented as fold change (n=10 in each group). n.s.p > 0.05, *** p < 0.001.

Downregulation of MBP mRNA levels in qRT-PCR assays was determined in EtOH- and tobacco exposed human FB-Es (Table 1B). Both tobacco and EtOH exposures were associated with synergistic downregulation of MBP mRNA expression (Figure 2A). Similar effects were found for BDNF (Figure 2B), when reduced levels of BDNF were determined in the same FB-Es. Strong downregulation of actin was found in tobacco-exposed FB-Es (Figure 2C).

### 2.3. Early exposure to EtOH and Tobacco associates with more dramatic downregulation of the MBP and BDNF mRNA expression in FB-Es, compared to late exposures in human FB-Es.

**Figure 3.**
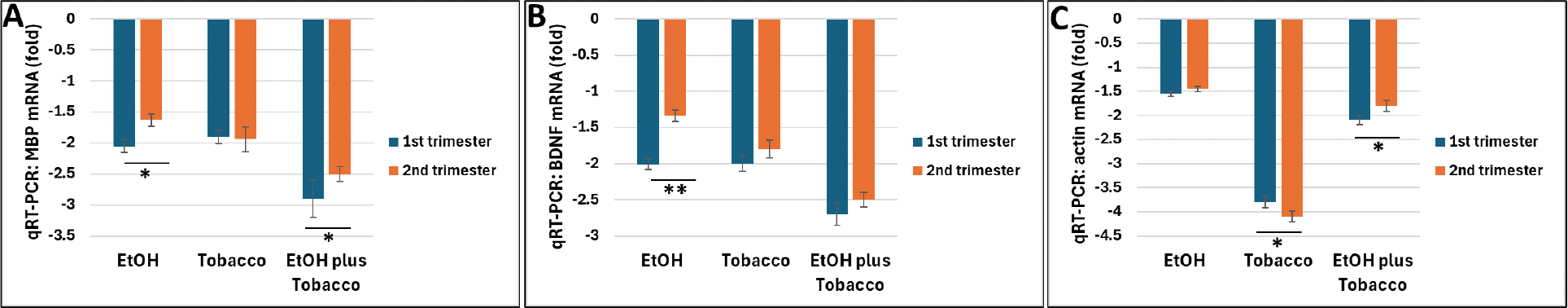
EtOH- and Tobacco-associated downregulation of the MBP, BDNF and actin mRNA expression in FB-Es from the 1st and the 2nd trimester samples. **A.** Downregulation of MBP levels in qRT-PCR assays in the same samples used for Figure 2. The strongest inhibition of MBP was seen in the 1st trimester EtOH cases or EtOH and Tobacco cases compared with the 2nd trimester EtOH- and EtOH plus tobacco-exposed FB-Es. **B.** Reduced levels of BDNF in 1st trimester cases in the same FB-Es. **C.** Downregulation of actin is greater in 2^nd^ trimester tobacco-exposed FB-Es. Data are presented as fold change. *p < 0.05, ** p < 0.01.

FB-Es from 1^st^ and 2^nd^ trimester blood samples were assayed for MBP, BDNF and actin (Figure 3). Downregulation of MBP levels was stronger in 1st trimester EtOH cases or EtOH and Tobacco cases compared with the 2nd trimester EtOH- and EtOH plus tobacco exposed FB-Es (Figure 3A). Reduced levels of BDNF were also strongest in 1st trimester cases in the same FB-Rs (Figure 3B), while downregulation of actin was stronger in the 2nd trimester tobacco-exposed FB-Es (Figure 3C).

### 2.4. MBP protein expression was significantly downregulated in EtOH and tobacco exposed FB-Es.

**Figure 4.**
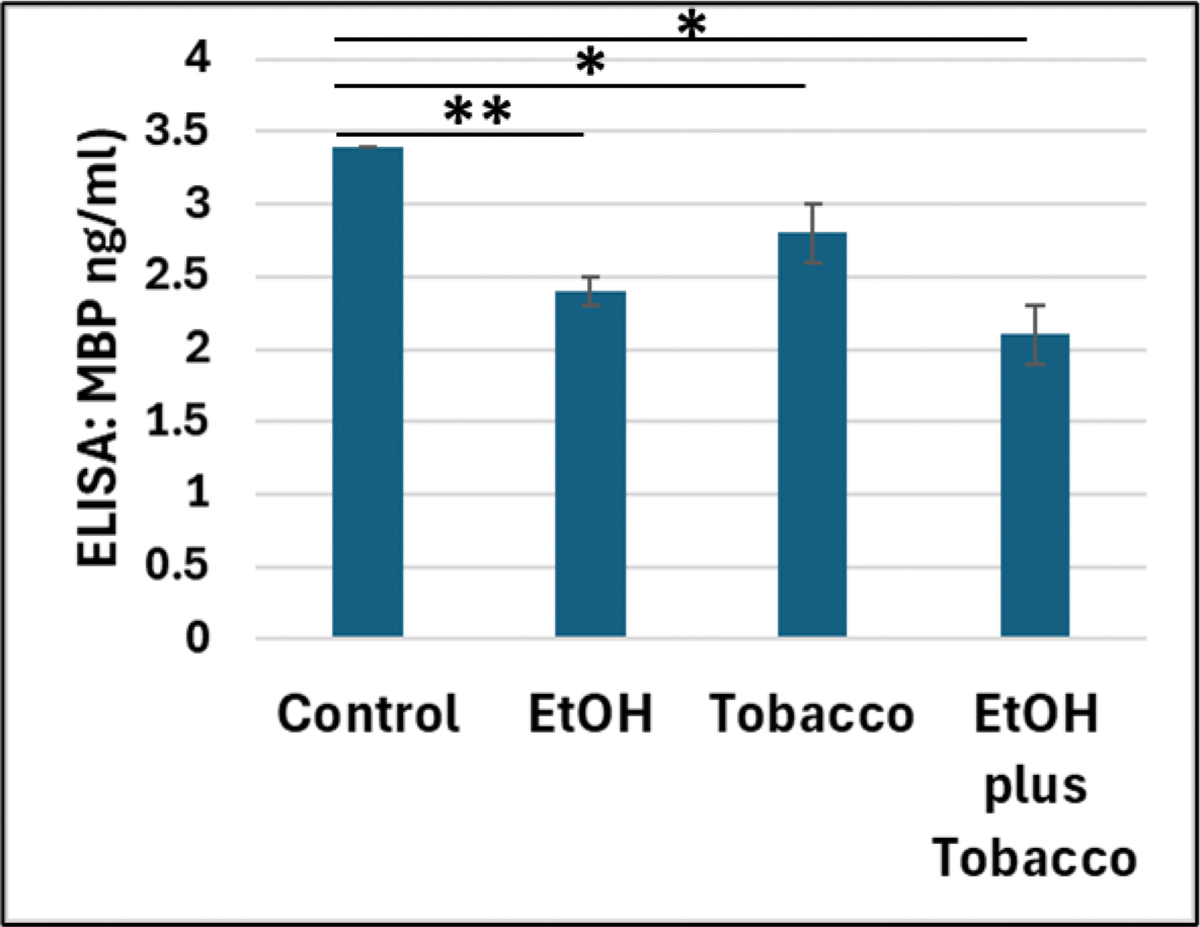
Downregulation of the MBP protein expression in EtOH- and tobacco-exposed FB-Es. Prenatal alcohol and tobacco exposure-associated downregulation of MBP protein in FB-Es was demonstrated by ELISA (ng/ml, according to MBP standards). All assays were done in triplicate. Detection range is within 0, 0.125, 0.25, 0.5, 1.0, 2.5, 5.0, 10ng/ml, or picogram/microliter, and sensitivity is 0.1ng/ml. * p < 0.05, ** p < 0.01.

Next, we confirmed our RNA findings for MBP with protein studies and demonstrated that the downregulation of MBP protein expression associated with prenatal alcohol and tobacco exposures are greater than that of either toxic factor alone (Figure 4).

### 2.5. Downregulation of MBP in EtOH-exposed oligodendrocyte-specific FB-Es (OL-Es).

**Figure 5:**
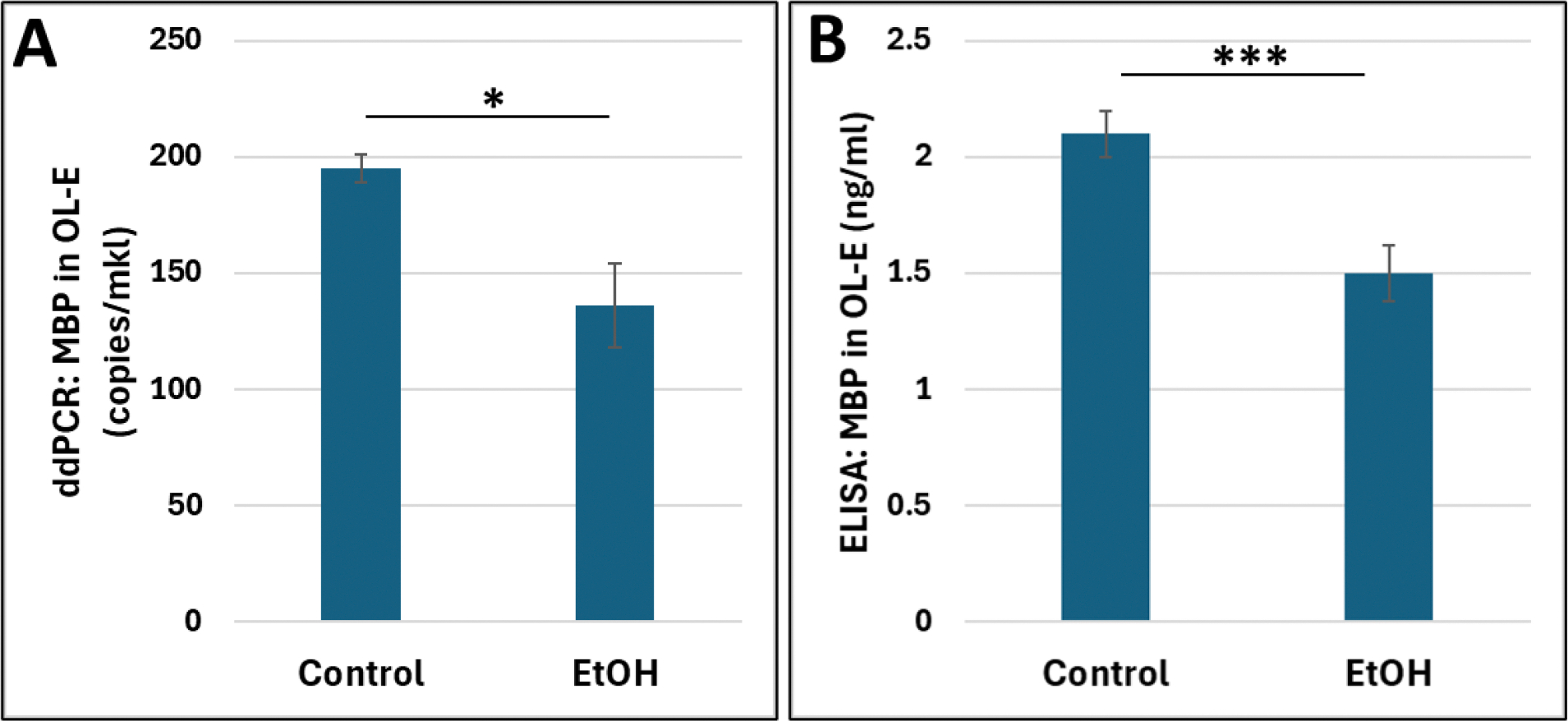
Downregulation of MBP mRNA and MBP protein expression in EtOH-exposed OL-Es. **A.** Impact of EtOH exposure on downregulation of MBP mRNA expression in OL-Es, from human FB-Es immunoprecipitated with the OL marker MBP (n=20) from 1st and 2nd trimesters was studied by ddPCR. Using OL-Es, we conducted studies of MBP mRNA and MBP protein expression **(B)**. The levels of MBP protein were analyzed in OL-Es using ELISA (ng/ml, according to MBP standards). All assays were done in triplicate. Detection range is within 0, 0.125, 0.25, 0.5, 1.0, 2.5, 5.0, 10ng/ml, or picogram/microliter, and sensitivity is 0.1ng/ml. The *p < 0.05, *** p < 0.001.

Finally, we isolated human OL-Es from 20 FB-Es, exposed to EtOH (Table 1C), to study the impact of EtOH exposure on downregulation of MBP in cell type-specific OL-Es (Figure 5). We found that both MBP mRNA (Figure 5A) and MBP protein (Figure 5B) were downregulated in EtOH cases compared to controls. Clinical characteristics of subjects used in the OL-E experiments are shown in Table 1C.

### 2.6. High BMI associates with strong downregulation of the MBP mRNA expression in EtOH- and tobacco-exposed FB-Es.

**Figure 6:**
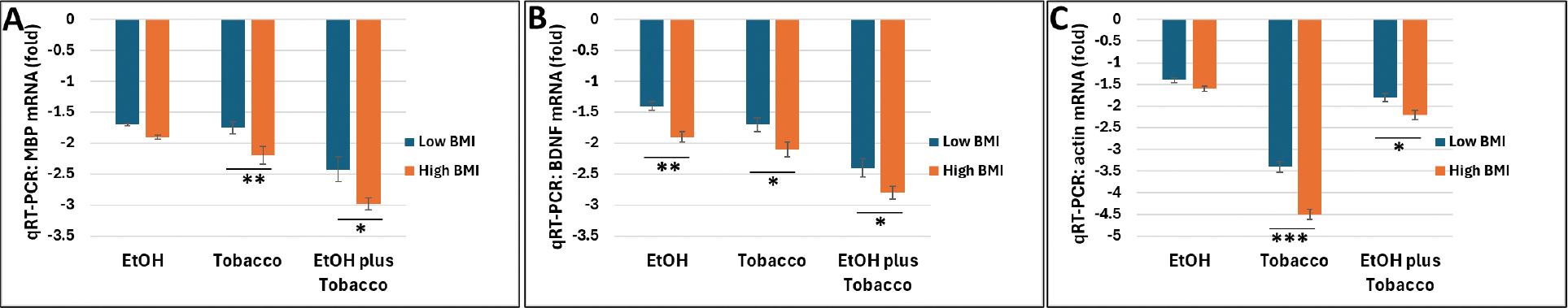
Downregulation of MBP mRNA expression in EtOH- and tobacco-exposed FB-Es associated with low or high BMI. **A.** Downregulation of MBP levels in qRT-PCR assays in the same samples used for Figure 2. Strongest inhibition of MBP was in EtOH cases FB-Es or in combination with high BMI compared to low BMI. **B.** Reduced levels of BDNF, strongest in all cases with high BMI in the same FB-Es. **C.** Downregulation of actin was strongest in high BMI tobacco-exposed FB-Es. Note that as in Figure 3C, the effect of tobacco alone is much greater than the effect of the combination of EtOH + tobacco. Data are presented as fold change. *p < 0.05, ** p < 0.01, *** p < 0.001.

Next, the effect of mObesity, or high BMI, was tested in FB-Es (Figure 6). MBP expression was downregulation in all cases with high BMI, compared to low BMI for all drug/health condition cases, or in combinations (Figure 6A). Similar effect of BMI on the level of expression of BDNF was found in all cases with one exposure, or both (Figure 6B), while actin was associated with lower levels in those with tobacco exposure in high BMI FB-Es (Figure 6C).

Thus, maternal EtOH and tobacco exposure, and even mObesity were associated with inhibition of the MBP protein not only in FB-Es, but also in cell type specific OL-Es.

## 3. Discussion

The present results show that maternal use of both EtOH and tobacco are associated with reductions in MBP levels in fetal brain, and in FB-Es, whereas other markers (actin) were affected differently by tobacco or EtOH exposures. MBP is integral to the process of myelination, which is key to the conduction of electrical impulses by axons.

### 3.1. Biomarkers for FASD.

FASD has been subdivided into several syndromes [1], of which the most severe, fetal alcohol syndrome (FAS), includes the full spectrum of craniofacial, somatic, and neurobehavioral abnormalities. We previously demonstrated that molecular abnormalities already are present early in fetuses exposed to EtOH [4]. These molecular abnormalities might reflect a direct effect of EtOH on the developing brain, or they might reflect transplacental movement of molecules associated with comorbid conditions in the mother. For example, maternal depression is seen frequently in pregnant women who drink EtOH. Our previous data suggested that maternal EtOH consumption and maternal depression, each independently and in combination, are associated with abnormalities of the epression at the mRNA and protein levels of several markers [4, 5]. Among these abnormalities was a dramatic downregulation of MBP expression in fetal brain and in FB-E. In the present study, exposure to EtOH or tobacco, as well as mDepression or mObesity, each was associated with downregulation of MBP in both brain tissues and FB-Es. The EtOH-associated changes in FB-Es were similar to those in fetal brain and may reflect molecular abnormalities that contribute to the pathogenesis of FASD.

### 3.2. Differential effects of EtOH vs. tobacco on actin expression.

Actin, a well-accepted housekeeping gene, has been used widely as a control for normalization of RNA or protein data. Here, we demonstrate that while alcohol exposure had little effect on actin expression, tobacco exposure strongly inhibited actin mRNA expression. Thus, early detection of actin could serve as a biomarker to predict the emergence of tobacco related disorders (cancers, heart diseases), while MBP abnormalities and low BDNF levels might serve as early biomarkers for FASD.

### 3.3. Synergistic effects of risk factors on expression of OL lineage biomarkers.

The effects of combinations of two risk factors on biomarker expression might show different patterns. 1) The combined effect might be smaller than or equal to the effect of either alone, perhaps by occluding signaling on converging downstream pathways. Such a pattern generally was not seen with combinations of EtOH, tobacco, mObesity or mDepression with regard to mRNA expression of molecular markers of OL development, although it did appear to affect actin expression, for which the inhibition appeared greater for tobacco alone than for tobacco + EtOH. 2) The effects of a combination might be greater than the effects of either alone, which might indicate that effects were additive, perhaps by affecting submaximal effects on the same or different pathways. This was the pattern seen with the effects of EtOH and tobacco on mRNA expression of BDNF and MBP. 3) The effects on biomarkers exceeds the sum of the individual effects, i.e., there is true synergy between the risk factors, suggesting that to some extent, one risk factor requires the presence of the second risk factor as a catalyst. In other contexts, this appears to be the case for the retinal damage in tobacco-alcohol amblyopia. In the present study, it appears to characterize the interactions between mObesity and either EtOH or mDepression. This striking finding warrants further investigation.

### 3.4. OL-specific FB-Es (OL-Es).

Previously, we developed a non-invasive method to investigate fetal brain proteins and RNAs by isolating FB-Es from maternal serum [12, 13]. In the present study, this strategy was further developed to isolate OL-Es to investigate MBP in the fetal brain. Maternal serum proteins have been used to predict infant outcomes and might be useful in classifying difficult-to-diagnose FASD subpopulations. However, the ability to isolate OL-Es non-invasively from maternal blood and analyze their cargos for MBP and other biomarkers, even in the first trimester, might prove more specific for predicting the emergence of neurodevelopmental abnormalities such as FASD in at-risk children.

### 3.5. Limitations.

The present study used fetal tissues and maternal blood samples from mothers who elected to terminate their pregnancies, with two important limitations: - 1) The numbers of fetuses that could be studied were relatively small; 2) there was no possibility of postnatal follow-up studies to determine the clinical outcomes in the offspring. Currently, we are performing a much larger study on non-interrupted pregnancies to determine which biomarkers best predict whether an at-risk fetus will go on to have one of the forms of FASD postnatally. A similar approach might be used for tobacco or other toxic exposures.

## Conclusions

Individually, fetal exposure to EtOH, tobacco, mObesity or mDepression have similar effects to impair MBP expression whose level is related to both CNS myelin damage and fetal eye diameter (an anatomical hallmark of FAS). When these exposures are combined the effects appear to be greater than those of the risk factors individually, and the effects of mObesity appear to be truly synergistic with EtOH and mDepression. Tobacco exposure was associated with downregulation of BDNF, and the effect was stronger when combined with EtOH, while the effects on actin expression were different. While actin was downregulated by exposure to tobacco more than to EtOH, the effect of the combination of exposures was much smaller than that of tobacco alone. Our unique biobank can be used to determine the effects of specific adverse exposures and may suggest novel therapeutic or prophylactic interventions to lessen the severity of FASD. Detection of MBP levels early in alcohol-exposed pregnancy might become a promising tool for early diagnosis of FASD.

## 4. Materials and Methods

### 4.1. Clinical recruitment.

Brain tissue and maternal serum was collected from women undergoing elective pregnancy termination under IRB protocol approved by Temple University (#21476: Early Gestation Alcohol Exposure: Mechanisms of Human Developmental Injury, PI Dr. Darbinian, Nune). A detailed questionnaire was used based on the NICHD PASS study [14]. Samples were collected between 9 and 23 weeks GA, as summarized in Table 1.

### 4.2. Isolation of Fetal Brain-Derived Exosomes (FB-Es) from Maternal Serum.

Human FB-Es were isolated as described previously [4, 12].

### 4.3. Isolation of Fetal Brain Oligodendrocyte-Derived Exosomes (OL-Es) from Maternal Serum.

Human FB-Es described above, and anti-MBP antibody were used to isolate OL-derived exosomes.

### 4.4. ELISA Quantification of Exosomal Proteins.

ELISA, a plate-based assay technique was used for detecting and quantifying MBP in FB-Es and OL-Es, using a human MBP ELISA kit (NeoBiolab, Cambridge, Massachusetts). CD81 (American Research Products-Cusabio) were quantified according to the suppliers’ instructions. ELISA data were statistically evaluated using Excel (Microsoft 365, software version 2404) and statistical analysis tools: CurveExpert for ELISA statistics (CUSABIO) or APP 96-well Plate Assay Data Analysis Software 5.0.apk (Cloud-Clone, Katy, TX, USA), available online.

### 4.5. RNA Preparation and qRT-PCR.

Total RNA, FB-E RNA, and OL-E RNA were isolated using the RNeasy kit (Qiagen, Valencia, CA) with on-column DNA digestion. The RT-PCR reaction was performed with 100 ng total RNA, using One-Step FAST RT-PCR Syber Green mix (Qiagen) on a StepOnePlus Real-Time PCR system thermo cycler. GAPDH and actin housekeeping genes were used for the normalization. PCR conditions were activation 95°C 5 min, PCR 45 cycles: 95°C 10 sec, 60°C 20 sec, 72°C 30 sec, melting curve (95–65°C), cool to 40°C 30 sec. For relative quantification, the expression level of genes was normalized to the housekeeping gene β-actin. Results were in arbitrary units. The primers were used: β- actin, S 5′-CTACAATGAGCTGCGTGTGGC-3′, AS 5′-CAGGTCCAGACGCAGGATGGC-3′.

### 4.6. Droplet Digital PCR (ddPCR).

For absolute quantitation of mRNA copies, ddPCR was performed using the QX200 ddPCR system. Fifty nanograms of human fetal total RNA was used with the 1st Strand cDNA Synthesis Kit (Qiagen, Valencia, CA, USA). After reverse transcription, the cDNA (300 dilution) aliquots were added to the BioRad master mix to conduct ddPCR (EvaGreen ddPCR Supermix, BioRad, Hercules, CA, USA). The absolute quantity of DNA per sample (copies/μL) was calculated using QuantaSoft Analysis Pro Software (AP) (Bio-Rad, Hercules, CA, USA) to analyze ddPCR data for technical errors (Poisson errors). The ddPCR protocol yields a linear dynamic range of detection between 1 and 100,000 target mRNA copies/μL. The ddPCR data were exported to Microsoft Excel (Microsoft 365) for further statistical analysis.

### 4.7. Statistical Analysis.

Statistical analysis was performed using SPSS Statistics from IBM Corp., released in 2017 for Windows, Version 25.0 (Armonk, NY, USA). All data are represented as the mean ± SD for all performed repetitions. Means were analyzed by a one-way ANOVA, with Bonferroni correction, where appropriate. Statistical significance was defined as p < 0.05. Sample numbers are indicated in the figure legends.

### 4.8. Ethics: Human Subjects.

Consenting mothers were enrolled at between 9- and 23-weeks GA, using an IRB protocol approved by Temple University. This protocol involved no invasive procedures other than routine care.

All procedures involving collection and processing of blood samples were done according to NIH Guidelines through a trained Study Coordinator. The de-identified log sheets contain an assigned accession number, the age, sex, ethnicity, and race of the patient. Except for an assigned accession number, no identification was kept on the blood samples.

#### Eligibility Criteria.

The blood and placenta samples were obtained according to NIH Guidelines through a trained Study Coordinator. Samples were collected regardless of sex, ethnicity, and race. Subjects were excluded if they had an active urinary tract infection on history, nitrates or WBCs on clinical UA; no prisoners; no adults who are cognitively impaired or physically unable to provide consent to participate; no patients with severe blood disorders (e.g., hemophilia).

#### Treatment Plan.

Each patient was asked to sign a separate consent form for research on blood and tissue samples. Blood obtained was processed for collection of serum. No invasive procedures were performed on the mother, other than those used in her routine medical care. Placenta tissues were processed for protein isolation.

#### Risk and Benefits.

There were very small risks of loss of privacy as with any research study in which protected health information is viewed. The samples were depersonalized before they were sent to the lab for analysis. There were no additional risks of blood sampling as this was only performed in subjects with clinically indicated venous access. There was little anticipated risk from obtaining 2–3 cc of blood, but a well-trained Study Coordinator collected all samples.

There was no direct benefit to the research subjects from participation, but there is significant potential benefit for the future FASD subjects and the general population. This research represents a reasonable opportunity to further the understanding, prevention, or alleviation of a serious problem affecting the health or welfare of FASD patients.

#### Informed Consent.

Consent forms were maintained by the Study Coordinator and were not sent to the investigator with the samples. The de-identified log sheets and IRB protocol were sent by the Study Coordinator to the Principal Investigator with each blood and tissue sample.

## Figures and Tables

**Table 1:** Maternal blood and fetal brain tissues used in studies. **A.** Fetal brain tissue samples from EtOH-exposed cases, mObesity, mDepression, and combinations (n=6 each group) were matched individually for fetal sex, GA, and maternal age. PCR for SRY gene on Y chromosome for sex determination was performed for cases and control samples. **B.** Maternal blood samples from EtOH-exposed cases (n=10), tobacco exposed (n=10), EtOH- and tobacco-exposed cases (n=10), and Controls (n=10) were matched individually for GA. **C.** Clinical characteristics of subjects by EtOH exposure (n=20) vs Controls (n=20) for OL-Es studies.

A. Subjects used in the experiments for OL markers (in fetal brains).		
	Gestational Age (weeks)	Subjects (number)
Control	9 – 32	6
Maternal EtOH	9 – 23	6
Maternal Obesity	10 – 22	6
Maternal Depression	10 – 22	6
Maternal EtOH plus Obesity	10 – 22	6
Maternal EtOH plus Depression	10 – 22	6
Maternal Depression plus Obesity	10 – 22	6

B. Clinical characteristics of fetal subjects used in the EtOH and tobacco experiments (in FB-E).				
	Control subjects (no EtOH, n=10)	EtOH-exposed subjects (n=10)	Tobacco-consuming subjects (no EtOH, n=10)	EtOH plus tobacco consuming subjects (n=10)
Maternal Age (years ±SD)	26.17 ± 2.15	22.34 ± 1.70	24.28 ± 1.12	23.26 ± 1.52
Gestational Age (weeks ±SD)	15.47 ± 1.33	15.16 ± 1.42	15.48 ± 2.19	15.27 ± 2.15
Race: White vs Black (%)	50 vs 50	50 vs 50	40 vs 60	40 vs 60
BMI (high BMI vs low BMI, %)	50 vs 50	50 vs 50	50 vs 50	50 vs 50

C. Clinical characteristics of subjects used in the OL-E experiments.		
	Controls (n=20)	EtOH Users (n=20)
Age (maternal, years ±SD)	24.15 ± 2.3	28.0 ± 2.7
Age (gestational, weeks ±SD)	14.92 ± 1.58	15.22 ± 1.6
Gestational age range (weeks)	9 to 23	9 to 23
BMI (Body Mass Index ±SD)	24.4 ± 3.3	26.1 ± 1.5
Hispanic Ethnicity (%)	15	15
Black vs White (%)	50	50
Fetal Sex (Male vs Female)	50	50

## Data Availability

This study collected demographic, behavioral, and laboratory data from normal, healthy women and from women who drank alcohol during pregnancy. Our research team supports all these activities and has developed a data-sharing plan. We also recognize that additional benefits from data sharing may arise in the future that are not apparent at this time, and we are prepared to work specifically with NIH in addressing all requests for raw data. At the present time, we have not deposited any of these raw data in an existing databank, but will make the data available to other investigators on request, in a manner consistent with NIH guidelines. Consistent with NIH policy, shared data will be rendered “free of identifiers that would permit linkages to individual research participants and variables that could lead to deductive disclosure of the identity of individual subjects” Intellectual property and data generated under this project will be administered in accordance with both University and NIH policies, including the NIH Data Sharing Policy and Implementation Guidance of 5 March 2003, and 0925–0001 and 0925–0002 (Rev 07/2022 through 01/31/2026). With this caveat observed, data will be made available to the NIH/NICHD/NIAAA. Sufficient identifiers will be provided to the NIH so that research participants can be assigned a Global Unique Identifier (GUID), which is a universal subject ID that protects personally identifiable information (PII). The NIH will be implementing a new specific policy regarding data sharing https://grants.nih.gov/grants/guide/notice-files/NOT-OD-21-014.html, as of 25 January 2023. We will adopt that policy also. Data will be also available at https://www.mdpi.com/ethics accessed on 1 January 2025)

## Abbreviations:

CSF: Cerebrospinal Fluid
CNS: Central Nervous System
ddPCR: digital droplet Polymerase Chain Reaction
ELISA: Enzyme-Linked Immunosorbent Assay
EtOH: Ethanol, Alcohol
FAS: Fetal Alcohol Syndrome
FASD: Fetal Alcohol Spectrum Disorder
FB-Es: fetal brain exosomes
GA: Gestation Age
GAPDH: Glyceraldehyde Phosphate Dehydrogenase
IRB: Institutional Review Board
MS: Multiple Sclerosis
NC: Nitro-Cellulose membranes
OL-Es: oligodendrocyte-derived exosomes
PAGE: Polyacrylamide Gel Electrophoresis
PAE: Prenatal Alcohol Exposure
qRT-PCR: quantitative Reverse Polymerase Chain Reaction
SRY: Sex-determining region of the Y chromosome

## References

[1] Hoyme HE; Kalberg WO; Elliott AJ; Updated Clinical Guidelines for Diagnosing Fetal Alcohol Spectrum Disorders. Pediatrics 138 (2016): e20154256. 27464676 10.1542/peds.2015-4256 PMC4960726

[2] Darbinian N & Selzer ME Oligodendrocyte pathology in fetal alcohol spectrum disorders. Neural regeneration research, 17 (2022): 497–502. 34380877 10.4103/1673-5374.314294 PMC8504395

[3] Darbinian N, Darbinyan A, Merabova N, Ethanol-mediated alterations in oligodendrocyte differentiation in the developing brain. Neurobiology of disease, 148 (2021): 105181. 33189883 10.1016/j.nbd.2020.105181 PMC7856167

[4] Darbinian N, Darbinyan A, Sinard J, Molecular Markers in Maternal Blood Exosomes Allow Early Detection of Fetal Alcohol Spectrum Disorders. Int J Mol Sci 24 (2023): 135. 10.3390/ijms24010135 PMC9820501 36613580

[5] Darbinian N; Merabova N; Tatevosian G; Biomarkers of Affective Dysregulation Associated with In Utero Exposure to EtOH. Cells 13 (2024): 2. 10.3390/cells13010002 PMC10778368 38201206

[6] Odendaal HJ, Steyn DW, Elliott A, Combined effects of cigarette smoking and alcohol consumption on perinatal outcome. Gynecologic and obstetric investigation, 67 (2009): 1–8. 18685256 10.1159/000150597 PMC8252129

[7] Machaalani R, Thawley M, Huang J, Effects of prenatal cigarette smoke exposure on BDNF, PACAP, microglia and gliosis expression in the young male mouse brainstem. Neurotoxicology 74 (2019): 40–46. 31121239 10.1016/j.neuro.2019.05.009

[8] Syed S, & Lioutas V Tobacco-alcohol amblyopia: a diagnostic dilemma. Journal of the neurological sciences, 327 (2013): 41–45. 23477666 10.1016/j.jns.2013.02.004

[9] Behbehani R, Sergott RC, Savino PJ. Tobacco-alcohol amblyopia: a maculopathy? Br J Ophthalmol 89 (2005): 1543–1544. 16234480 10.1136/bjo.2005.079137 PMC1772913

[10] Goodwin RD, Ganz O, Weinberger AH, Menthol Cigarette Use Among Adults Who Smoke Cigarettes, 2008–2020: Rapid Growth and Widening Inequities in the United States. Nicotine Tob Res 25 (2023): 692–698. 36223889 10.1093/ntr/ntac214 PMC10216874

[11] Ou X, Thakali KM, Shankar K, Maternal adiposity negatively influences infant brain white matter development. Obesity (Silver Spring) 23 (2015): 1047–1054. 25919924 10.1002/oby.21055 PMC4414042

[12] Goetzl L, Darbinian N, Goetzl EJ. Novel window on early human neurodevelopment via fetal exosomes in maternal blood. Ann Clin Transl Neurol 3 (2016): 381–385. 27231707 10.1002/acn3.296 PMC4863750

[13] Goetzl L, Darbinian N, Merabova N. Noninvasive assessment of fetal central nervous system insult: Potential application to prenatal diagnosis. Prenat Diagn 39 (2019): 609–615. 31069822 10.1002/pd.5474

[14] Spong CY, Mercer BM, D’Alton M, Timing of indicated late-preterm and early-term birth. Obstet Gynecol 118 (2011): 323–333. 21775849 10.1097/AOG.0b013e3182255999 PMC3160133

